# Effectiveness and selection of exercise prescriptions for myocardial infarction patients: a systematic review and meta-analysis

**DOI:** 10.3389/fcvm.2026.1739046

**Published:** 2026-02-23

**Authors:** Shiguang Ren, Yinping Zeng, Kun Qin, Yunzhu Hua, Jae Cheol Kim, Wenduo Liu, Zilin Wang

**Affiliations:** 1 College of Physical Education, Beihua University, Jilin, China; 2 Department of Sports Science, College of Natural Science, Jeonbuk National University, Jeonju, Republic of Korea

**Keywords:** exercise, myocardial infarction, rehabilitation, acute coronary syndrome (STEMI, NSTEMI), left ventricular ejection fraction

## Abstract

**Background:**

Exercise intervention has garnered significant attention for its potential to promote recovery and improve outcomes in myocardial infarction (MI) patients. However, controversy persists regarding the efficacy of exercise prescriptions in related studies. Therefore, this research aims to analyze the effects of exercise prescriptions during MI rehabilitation and associated influencing factors through systematic review and meta-analysis, thereby providing guidance for exercise prescription selection in MI patients.

**Methods:**

A systematic review and meta-analysis were conducted by retrieving data from PubMed, Web of Science, and Scopus databases between January 2015 and June 2025. Only meta-analyses using random-effects models for cardiac function were included in the study. Subgroup analyses were conducted based on exercise type, intensity, session duration, frequency, and intervention duration.

**Results:**

A total of 42 studies were included. The results showed that exercise intervention significantly improves cardiac function, exercise performance, and quality of life in MI patients, while significantly reducing the incidence of adverse cardiovascular events. A subgroup analysis of 26 cardiac function studies revealed that variations in exercise type, intensity, frequency, session duration, and intervention duration all exerted modulatory effects on left ventricular ejection fraction (LVEF) levels in MI patients. However, the certainty of evidence related to LVEF is generally low, and the pooled effect is mainly influenced by non-randomized studies with high risk of bias. Furthermore, the evidence is subject to inconsistency and/or imprecision, leading to a potential low or very low certainty in conclusions.

**Conclusion:**

Current findings indicate that moderate-intensity aerobic or resistance training, with each session lasting < 30 min, < 3 times per week, and an intervention duration > 16 weeks, is more beneficial for the rehabilitation of MI patients. The clinical research on resistance training is currently insufficient, and some studies have a high risk of bias. Additionally, there is considerable heterogeneity in the heart function intervention methods (e.g., type, session duration, frequency, intensity, and intervention duration), which could potentially influence the research outcomes. Therefore, more high-quality studies are needed in the future to validate these findings and provide more reliable scientific evidence for optimizing rehabilitation strategies for MI patients.

**Systematic Review Registration:**

https://www.crd.york.ac.uk/PROSPERO/view/CRD420251085069, PROSPERO CRD420251085069.

## 1 Introduction

Myocardial infarction (MI) is one of the primary acute coronary events leading to sudden cardiac death (1). Its pathogenesis is primarily associated with the instability of atherosclerotic plaques in the coronary arteries (e.g., rupture or erosion), which in turn trigger platelet aggregation and the coagulation cascade, resulting in thrombus formation (2). This subsequently causes acute coronary artery occlusion, resulting in severe and persistent myocardial ischemia, ultimately culminating in myocardial cell necrosis (3). Clinically, it manifests primarily as persistent severe chest pain, palpitations, dyspnea, diaphoresis, nausea, and other autonomic symptoms. In severe cases, patients may experience cardiogenic shock, life-threatening arrhythmias, or even sudden cardiac death (4, 5). Currently, clinical monitoring of cardiac function recovery after MI primarily relies on left ventricular ejection fraction (LVEF) as the core indicator for assessing ventricular pumping capacity and prognosis. Decreased LVEF typically reflects myocardial injury and ventricular dysfunction, and is closely associated with rehospitalization rates, heart failure, and mortality risk (6). Studies indicate that when LVEF falls below 35%, patient mortality and complication risks significantly increase (7). Consequently, improving LVEF has emerged as a pivotal objective in post-MI rehabilitation.

In recent years, exercise interventions have been widely applied in the rehabilitation of patients with MI. Studies indicate that aerobic exercise (AE), resistance training (RE), and Compound Exercise (CE; AE + RE) can effectively improve cardiopulmonary function and exercise endurance in MI patients (8, 9). Mechanistically, exercise activates AMP-activated protein kinase (AMPK), upregulates genes involved in endogenous antioxidant defenses, and promotes peroxisome proliferator-activated receptor gamma coactivator 1-alpha–mediated mitochondrial adaptations, thereby strengthening antioxidant systems such as manganese superoxide dismutase, copper/zinc superoxide dismutase, and catalase. Exercise may also suppress angiotensin II (Ang II) expression and nicotinamide adenine dinucleotide phosphate oxidase activity—both closely associated with reactive oxygen species (ROS) generation—via the sirtuin 1/AMPK/nuclear factor erythroid 2-related factor 2 axes, ultimately reducing ROS production (10). Meanwhile, exercise-induced skeletal muscle–derived interleukin-6 can stimulate the production of anti-inflammatory mediators, including interleukin-1 receptor antagonist and interleukin-10 (IL-10), while inhibiting the expression of pro-inflammatory cytokines such as tumor necrosis factor alpha (TNF-α) and IL-1β. Accordingly, post-training inflammatory markers decrease and the IL-10/TNF-α ratio improves, further reinforcing anti-inflammatory effects (11). In addition, myocardial infarction is often accompanied by autonomic imbalance characterized by heightened sympathetic activity and reduced vagal tone, this imbalance interacts bidirectionally with immune activation and persistent inflammation, potentially creating a vicious cycle. Exercise training can restore autonomic balance (e.g., increased heart rate variability), thereby attenuate sustained inflammatory responses and providing a physiological basis for favorable ventricular structural and functional remodeling (11). Furthermore, the efficacy of exercise interventions in alleviating symptoms associated with anxiety and depression has been demonstrated (12), enhancing quality of life and reducing both hospitalization rates and mortality (13). Although exercise interventions have shown potential benefits in MI rehabilitation, substantial heterogeneity exists across studies in key prescription parameters—such as intensity, frequency, session duration, and intervention duration. Prior systematic reviews have largely focused on summarizing overall effects (9), with limited efforts to synthesize optimal parameter combinations in a structured manner. This gap contributes to inconsistent evidence and, consequently, undermines the generalizability of intervention effects and the precision of their clinical application.

This study aimed to investigate whether exercise-based rehabilitation programs (I), compared with no exercise or usual/standard care (C), improve cardiac function, exercise performance, and quality of life, and reduce adverse events (O) in patients with MI (P). Eligible studies (S) included randomized controlled trials (RCT), quasi-experimental study, retrospective observational study, retrospective cohort study, and observational cohort study. Systematically evaluates the impact of exercise prescriptions on rehabilitation outcomes in patients with MI through meta-analysis and subgroup analysis of cardiac function indicators. The study goes on to explore the moderating effects of different exercise prescription components on rehabilitation efficacy. This provides scientific evidence for the optimization of exercise prescriptions and the enhancement of clinical practice for patients suffering from MI.

## 2 Materials and methods

### 2.1 Protocol and registration

The present study adheres to the Preferred Reporting Items for Systematic Reviews and Meta-Analyses 2020 (PRISMA 2020) guidelines for conducting systematic reviews and meta-analyses (14), and has been registered on the International Prospective Registration Platform for Systematic Reviews (PROSPERO; Registration number: CRD420251085069).

### 2.2 Search strategy

The present study conducted a systematic search across three major databases: PubMed, Web of Science, and Scopus. The search period was restricted to January 2015 through June 2025 to obtain the latest research advances and clinical evidence (Table 1). The retrieval of English-language literature was conducted using the following keywords and their synonyms: “exercise”, “myocardial infarction (MI)”, “rehabilitation”, “acute coronary syndrome (ACS)”, “ST-segment elevation myocardial infarction (STEMI)”, and “non–ST-segment elevation myocardial infarction (NSTEMI)”. The employment of Boolean operators (AND, OR) during the search process ensured the combination of keywords, thus facilitating the identification of both comprehensive and relevant literature.

**Table 1:** Search strategy.

PubMed (January 2015 through June 2025)
([“Myocardial Infarction”(Mesh) OR “MI”OR “Acute Myocardial Infarction” OR “Cardiac Infarction”]OR[“Acute Coronary Syndrome”(Mesh) OR “ACS”] AND [“Rehabilitation"(Mesh) OR “Functional Recovery” OR “Physical Rehabilitation” OR “Therapeutic Recovery” OR “Cardiac Rehabilitation” OR “Physical Recovery” OR “Recovery Therapy” OR “Rehabilitation Training”] AND [“Exercise”(Mesh) OR “Physical Activity” OR “Aerobic Exercise” OR “Resistance Training” OR “Physical Training” OR “High-Intensity Interval Training” OR “Moderate-Intensity Interval Training”])
Web of Science (January 2015 through June 2025)
TS = [(“Myocardial Infarction” OR “MI” OR “Acute Myocardial Infarction” OR “Cardiac Infarction” OR “Acute Coronary Syndrome” OR “ACS”) AND(“Rehabilitation” OR “Functional Recovery” OR “Physical Rehabilitation” OR “Therapeutic Recovery” OR “Cardiac Rehabilitation” OR “Physical Recovery” OR “Recovery Therapy” OR “Rehabilitation Training”)AND(“Exercise” OR “Physical Activity” OR “Aerobic Exercise” OR “Resistance Training” OR “Physical Training” OR “High-Intensity Interval Training” OR “Moderate-Intensity Interval Training”))
Scopus (January 2015 through June 2025)
TITLE-ABS-KEY [(“Myocardial Infarction” OR “MI” OR “Acute Myocardial Infarction” OR “Cardiac Infarction” OR “Acute Coronary Syndrome” OR “ACS”) AND (“Rehabilitation” OR “Functional Recovery” OR “Physical Rehabilitation” OR “Therapeutic Recovery” OR “Cardiac Rehabilitation” OR “Physical Recovery” OR “Recovery Therapy” OR “Rehabilitation Training”)AND(“Exercise” OR “Physical Activity” OR “Aerobic Exercise” OR “Resistance Training” OR “Physical Training” OR “High-Intensity Interval Training” OR “Moderate-Intensity Interval Training”))

### 2.3 Inclusion and exclusion criteria

In accordance with the PICOS framework, studies must fulfil the following criteria for inclusion in the analysis: (1) Population (P): Patients with MI (STEMI/NSTEMI); (2) Intervention (I): Exercise as the primary intervention; (3) Comparison (C): Control groups receiving alternative interventions or no intervention; (4) Outcomes (O): outcome measures included changes in cardiac function (LVEF), exercise performance (HR, BP, MET, VO2max, AT, Endurance exercise capacity, Gait test), and quality of life (SF-36, MacNew, QLMI, SAQ, CROQ, EQ-5D, KASI, WHOQOL-BREF), as well as changes in the incidence of adverse cardiovascular events (including cardiac death, MI recurrence, rehospitalization, arrhythmias, etc.); (5) Study design (S): RCT, quasi-experimental study, retrospective observational study, retrospective cohort study, and observational cohort study.

The following criteria were used to determine exclusion from the study: (1) Animal studies; (2) Studies not employing exercise as an intervention; (3) Non-controlled or non-interventional studies; (4) Article types including reviews, conference abstracts, case reports, and surveys; (5) Articles where full text is unavailable or not written in English; (6) Studies reporting only the overall ACS population without providing independent data for MI (STEMI/NSTEMI) or clear diagnostic criteria.

### 2.4 Quality assessment and risk of bias

Two authors (S.R. and Y.Z.) independently assessed the quality of the literature and identified risk of bias by using the mixed methods appraisal tool (MMAT) (15) and the Cochrane risk of bias assessment tools (ROB 2.0 and ROBINS-I) (16, 17). Meanwhile, the certainty of evidence was graded using the GRADE approach (18). The GRADE certainty was rated down one (serious concern) or two grades (very serious concern) for reasons including risk of bias, inconsistency, indirectness, imprecision, and publication bias (18). ROB 2.0 was primarily utilized for the evaluation of bias risk in randomized controlled trials (RCTs), while ROBINS-I was employed for non-RCTs. The risk of bias in each domain was categorized as “low risk of bias”, “high risk of bias”, or “unclear risk of bias” according to the recommendations of the Cochrane Handbook. In instances of discordance between the two assessors, a third author (W.L.) was consulted to facilitate discussion and reach a final consensus. To evaluate inter-rater reliability, Cohen's kappa statistic was calculated to assess the level of agreement between reviewers.

### 2.5 Data extraction and analysis

The retrieved literature was downloaded into Endnote software, with the process of removing duplicate entries. The selection process was conducted by two authors (S.R. and Y.Z.) who independently reviewed the abstracts and full texts of studies that met the inclusion criteria. A comprehensive evaluation of the eligible studies was conducted, encompassing the following aspects: (1) the identity of the primary author; (2) the year of publication; (3) the study design; (4) the sample size of the study; (5) the age of the subject; (6) the intervention measures (type of exercise, intensity, frequency, session duration, intervention duration); (7) the control measures; and (8) the outcome measures (changes in cardiac function, exercise endurance, quality of life, and incidence of adverse cardiovascular events).

### 2.6 Statistical analysis

This meta-analysis was performed using Review Manager (RevMan) version 5.4. All extracted outcomes were continuous variables. Because the outcome measures were consistent across the included studies, pooled effects were calculated using the weighted mean difference (WMD) with 95% confidence intervals (CI). A DerSimonian–Laird random-effects model was applied to estimate the between-study variance (*Tau²*) and to synthesize the results. Statistical heterogeneity was assessed using the *I²* statistic, with *I²* values of 25%–50% indicating low heterogeneity, 50%–75% indicating moderate heterogeneity, and >75% indicating substantial heterogeneity. The pooled results were presented as forest plots. Publication bias was evaluated using funnel plots and Egger's regression test. Prespecified subgroup analyses were conducted according to intervention type, intensity, frequency, duration, and intervention period. Subgroup interaction was assessed using the between-subgroup heterogeneity Q test (*χ²* test; test for subgroup differences), and the Q statistic, degrees of freedom (df), and *P* value were reported.

## 3 Results

### 3.1 Study selection

**Figure 1:**
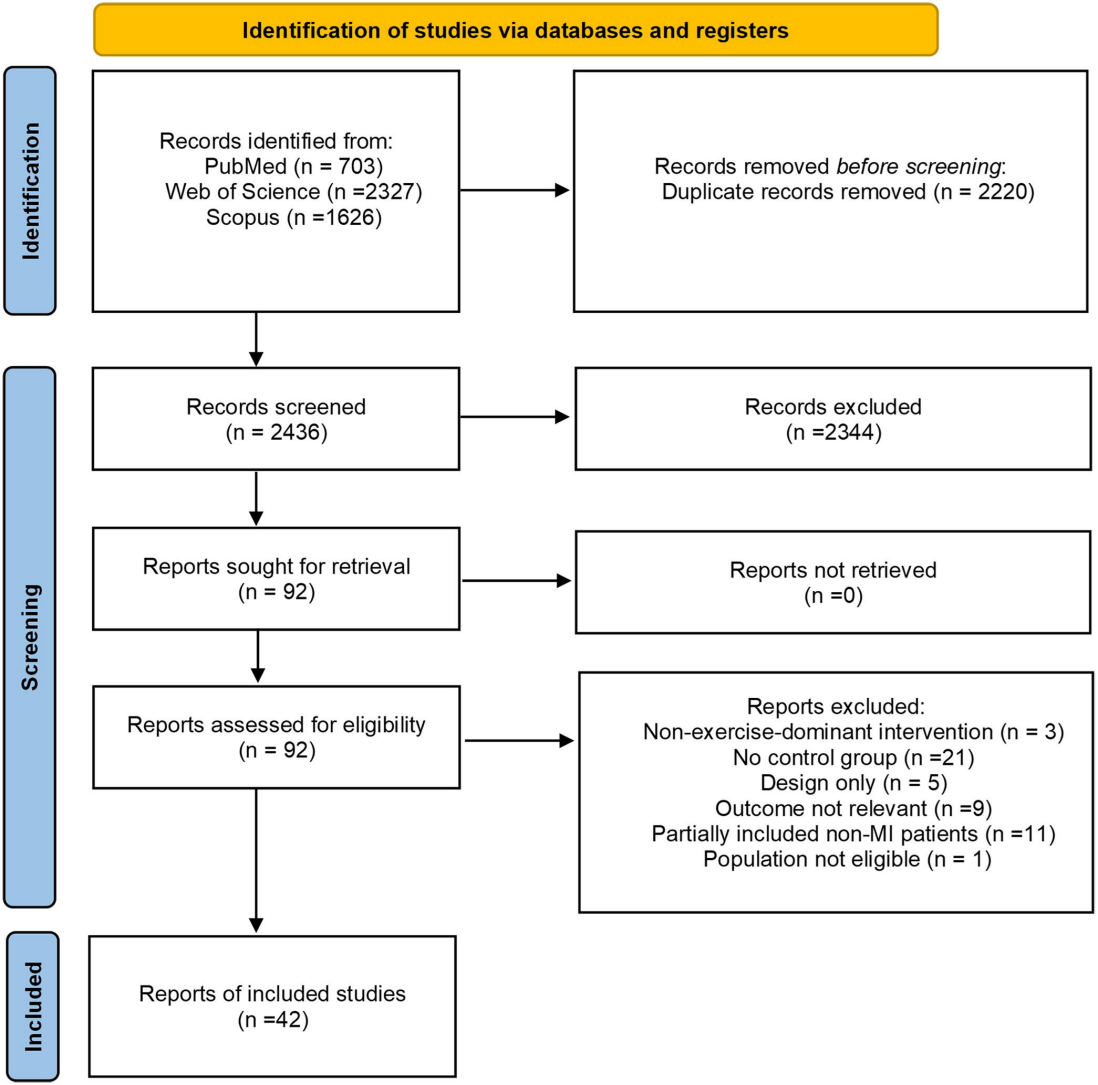
PRISMA flow diagram of the study.

A comprehensive search of the literature was conducted using three major bibliographic databases: PubMed, Web of Science, and Scopus. The results of this search yielded 703, 2,327, and 1,626 articles, respectively. Following the removal of 2,220 duplicates, 2,436 records were subjected to a screening process to identify those that met the inclusion criteria. This screening involved an examination of the titles and abstracts of the records. Subsequently, a total of 42 studies were selected for inclusion in the systematic review, while 50 studies were excluded on the basis that they did not meet the stipulated inclusion criteria. Excluded studies included review articles, research design studies, animal experiments, studies without a control group, studies involving subjects other than MI patients, interventions not primarily exercise-based, studies with outcomes not meeting the inclusion criteria, and studies not published in English. As illustrated by Figure 1, the PRISMA flow diagram details the search process.

### 3.2 Basic characteristics of included studies

A total of 42 studies were included, comprising 25 RCTs and 17 non-RCTs, with a total of 8,083 participants included for further analysis. The basic characteristics of the included studies, including the first author, publication year, study design, sample size, age, intervention methods (type of exercise, intensity, frequency, session duration, and Intervention duration), progression, control group interventions, and outcome measures, are summarized in Supplementary Table S1.

### 3.3 Quality assessment

The present study employed mixed methods appraisal tool (MMAT) to evaluate the quality of the included literature. The MMAT assessment encompasses five research types: qualitative studies, quantitative randomized controlled trials, quantitative non-randomized studies, quantitative descriptive studies, and mixed-method studies. Prior to formally conducting the methodological quality assessment, the MMAT first established two universal screening questions: (1) Does the study clearly state its research question? (2) Is the data collected sufficient to answer the research question? It is important to note that only studies that provided a positive response to both screening questions proceeded to the in-depth methodological quality assessment. This involved the application of five criteria specific to each study design type, with each criterion assessed as “Yes”, “No”, or “Can't tell” (15). In accordance with MMAT recommendations, the assignment of an overall “total score” or the categorization literature into “high-medium-low” quality grades is not advised. Consequently, as illustrated in Table 2, the assessment does not provide an aggregate score during quality assessment. Instead, it presents the fulfillment status for each criterion individually to maintain objectivity and transparency in the evaluation. The inter-rater agreement for MMAT quality assessment was evaluated using Cohen's kappa (*κ* = 0.83), indicating strong agreement between the two reviewers.

**Table 2:** Quality assessment using mixed-methods appraisal tools (*n* = 42).

Number	References	Screening S1	Screening S2	Type of study	Q1	Q2	Q3	Q4	Q5
1	Fontes-Carvalho et al., (19)	Yes	Yes	Quantitative randomized controlled trials	Yes	Yes	Yes	Yes	Yes
2	Omiya et al., (20)	Yes	Yes	Quantitative non-randomized	Yes	Yes	Yes	Can't tell	Yes
3	Izeli et al., (21)	Yes	Yes	Quantitative non-randomized	Yes	Yes	Yes	Can't tell	Yes
4	Gloc et al., (22)	Yes	Yes	Quantitative randomized controlled trials	Yes	Yes	Yes	Yes	Yes
5	Lee et al., (23)	Yes	Yes	Quantitative non-randomized	Yes	Yes	no	Can't tell	Yes
6	Santi et al., (24)	Yes	Yes	Quantitative randomized controlled trials	Yes	Yes	Yes	Can't tell	Yes
7	Kunjan et al., (25)	Yes	Yes	Quantitative non-randomized	Can't tell	Yes	Can't tell	no	Yes
8	McGregor et al., (26)	Yes	Yes	Quantitative non-randomized	Yes	Yes	Yes	Can't tell	Yes
9	Zhang et al., (27)	Yes	Yes	Quantitative randomized controlled trials	Yes	Yes	Yes	Can't tell	Yes
10	Farheen et al., (28)	Yes	Yes	Quantitative randomized controlled trials	Yes	Yes	Yes	Can't tell	Yes
11	Trachsel et al., (29)	Yes	Yes	Quantitative randomized controlled trials	Yes	Yes	Yes	Yes	Yes
12	Ul-Haq et al., (30)	Yes	Yes	Quantitative randomized controlled trials	Yes	Can't tell	Yes	no	no
13	Ma et al., (31)	Yes	Yes	Quantitative non-randomized	Yes	Yes	Yes	no	Can't tell
14	Nowak et al., (32)	Yes	Yes	Quantitative randomized controlled trials	Yes	Yes	Yes	Can't tell	Yes
15	Cai et al., (33)	Yes	Yes	Quantitative non-randomized	Yes	Yes	Yes	Yes	Yes
16	Cai et al., (34)	Yes	Yes	Quantitative randomized controlled trials	Yes	Yes	Yes	Can't tell	Yes
17	Dehghani et al., (35)	Yes	Yes	Quantitative randomized controlled trials	Yes	Yes	Yes	Yes	Yes
18	Gloc et al., (36)	Yes	Yes	Quantitative randomized controlled trials	Yes	Yes	Yes	Can't tell	Yes
19	Lee et al., (37)	Yes	Yes	Quantitative non-randomized	Yes	Yes	Yes	Can't tell	Yes
20	Ma et al., (38)	Yes	Yes	Quantitative non-randomized	Yes	Yes	Yes	Yes	Can't tell
21	Wang et al., (39)	Yes	Yes	Quantitative randomized controlled trials	Yes	Yes	Yes	Can't tell	Yes
22	Bolatbekov et al., (40)	Yes	Yes	Quantitative non-randomized	Yes	Yes	Yes	Can't tell	Yes
23	Choi et al., (41)	Yes	Yes	Quantitative non-randomized	Yes	Yes	Yes	Yes	Yes
24	Elshazly et al., (42)	Yes	Yes	Quantitative randomized controlled trials	Yes	Yes	Yes	Can't tell	Yes
25	Eser et al., (43)	Yes	Yes	Quantitative randomized controlled trials	Yes	Yes	Yes	Yes	Yes
26	Kambic et al., (44)	Yes	Yes	Quantitative randomized controlled trials	Yes	Yes	Yes	Can't tell	Yes
27	Nowak-Lis et al., (45)	Yes	Yes	Quantitative randomized controlled trials	Yes	Yes	Yes	Can't tell	Yes
28	Qu et al., (46)	Yes	Yes	Quantitative non-randomized	Can't tell	Yes	Yes	Can't tell	Yes
29	Aispuru-Lanche et al., (47)	Yes	Yes	Quantitative randomized controlled trials	Yes	Yes	Yes	Yes	Yes
30	Lima et al., (48)	Yes	Yes	Quantitative randomized controlled trials	Yes	Yes	Yes	Yes	Yes
31	Aispuru-Lanche et al., (49)	Yes	Yes	Quantitative randomized controlled trials	Yes	Yes	Yes	Yes	Yes
32	Hiruma et al., (50)	Yes	Yes	Quantitative non-randomized	Yes	Yes	Yes	Yes	Yes
33	Hou et al., (51)	Yes	Yes	Quantitative non-randomized	Yes	Yes	Can't tell	Yes	Yes
34	Jo et al., (52)	Yes	Yes	Quantitative randomized controlled trials	Yes	Yes	no	Can't tell	Can't tell
35	Ksela et al., (53)	Yes	Yes	Quantitative randomized controlled trials	Yes	Yes	Yes	Can't tell	Yes
36	Liang et al., (54)	Yes	Yes	Quantitative non-randomized	Yes	Yes	Can't tell	no	Yes
37	Mitropouloset al., (55)	Yes	Yes	Quantitative randomized controlled trials	Yes	Yes	Yes	Yes	Yes
38	Yoon et al., (56)	Yes	Yes	Quantitative non-randomized	Can't tell	Yes	no	Yes	Yes
39	Hou et al., (57)	Yes	Yes	Quantitative non-randomized	Yes	Yes	Yes	Yes	Yes
40	Nowak-Li et al., (58)	Yes	Yes	Quantitative randomized controlled trials	Yes	Yes	Yes	Can't tell	Yes
41	Zhao et al., (59)	Yes	Yes	Quantitative randomized controlled trials	Yes	Yes	Yes	Yes	Yes
42	Zhao et al., (60)	Yes	Yes	Quantitative randomized controlled trials	Yes	Yes	Yes	Yes	Yes

Screening S1: Are there clear research questions? Screening S2: Do the collected data allow to address the research questions? Quantitative randomized controlled trials: Q1. Is randomization appropriately performed? Q2. Are the groups comparable at baseline? Q3. Are there complete outcome data? Q4. Are outcome assessors blinded to the intervention provided? Q5. Did the participants adhere to the assigned intervention? Quantitative non-randomized: Q1. Are the participants representative of the target population? Q2. Are measurements appropriate regarding both the outcome and intervention (or exposure)? Q3. Are there complete outcome data? Q4. Are the co-founders accounted for in the design and analysis? Q5. During the study period, is the intervention administered (or exposure occurred) as intended?.

Quality of evidence for the LVEF outcomes was evaluated using the GRADE approach (restricted to the 26 LVEF outcomes only). A summary of findings table is presented in Supplementary Table S2. The overall certainty of the evidence is low: 2 studies are rated as moderate, 9 as low, and 15 as very low. Since the included evidence mainly comes from non-randomized studies, many of which have a high risk of bias, the pooled effect estimate is largely influenced by these studies. Additionally, there is inconsistency and/or indirectness, leading to the majority of the conclusions being rated as low or very low in certainty.

### 3.4 Risk of bias assessment

The present study systematically assessed the risk of bias in 42 including studies using the RoB 2.0 and ROBINS-I tools. Specifically, the RoB 2.0 was applied to evaluate the risk of bias in 25 RCTs, while ROBINS-I was utilized to assess the risk of bias in 17 non-RCT studies.

**Figure 2:**
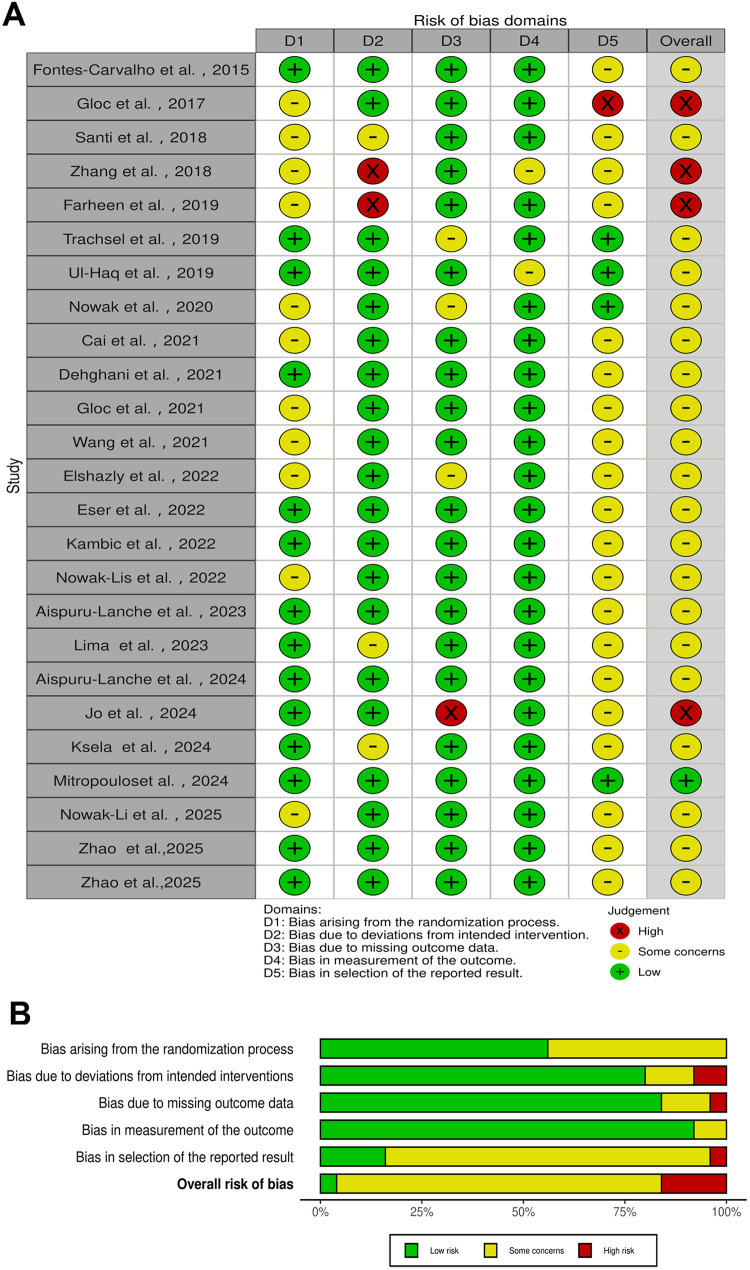
RCT ROB 2.0 risk of bias graph. **(A)** Domain-level judgments; **(B)** distribution of risk-of-bias judgments within each bias domain.

As illustrated in Figure 2, 4 of the 25 RCTs were found to be at high risk of bias, 1 study was found to be at low risk of bias, and the remaining 20 studies moderate raised concerns about bias. Within the first domain, some studies failed to explicitly report allocation concealment methods, introducing uncertainty in the assessment. As demonstrated in Figure 2, 2 studies exhibited high risk of bias in the second domain. This high risk of bias may be attributable to the nature of the intervention and limitations in the study setting. Additionally, 1 study demonstrated high risk of bias in outcome data, while another study indicated a high risk of bias in selective reporting of results. The inter-rater agreement for the RoB 2.0 risk of bias assessment was evaluated using Cohen's kappa (*κ* = 0.74), indicating substantial agreement between the two reviewers.

**Figure 3:**
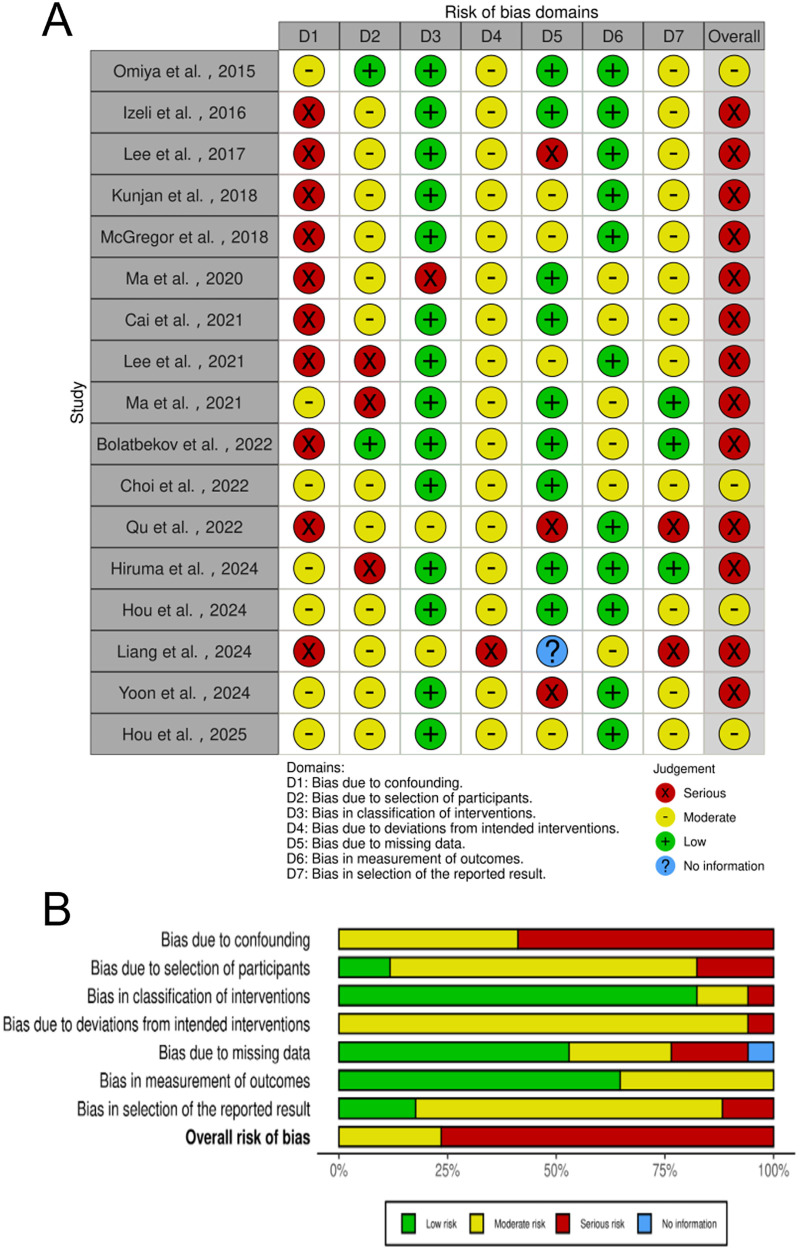
Non-RCT ROBINS-I risk of bias graph. **(A)** Domain-level judgments; **(B)** distribution of risk-of-bias judgments within each bias domain.

Among the 17 non-RCT studies, Figure 3 details the risk of bias assessment for each study using the ROBINS-I tool. 4 studies exhibited moderate risk of bias, and 13 studies exhibited high risk of bias. Confounding factors constitute one of the primary sources of bias in non-randomized studies. In certain investigations, the study design proved inadequate in controlling for the interaction between “self-selection” or “self-referral” mechanisms and variables such as socioeconomic status and health awareness. This made it difficult to clearly distinguish the true causal relationship between the intervention and health outcomes, leading to a significantly increased risk of confounding bias. The inter-rater agreement for the ROBINS-I risk of bias assessment was evaluated using Cohen's kappa (*κ* = 0.77), indicating substantial agreement between the two reviewers.

### 3.5 Regulatory role of exercise intervention on rehabilitation in MI patients

**Figure 4:**
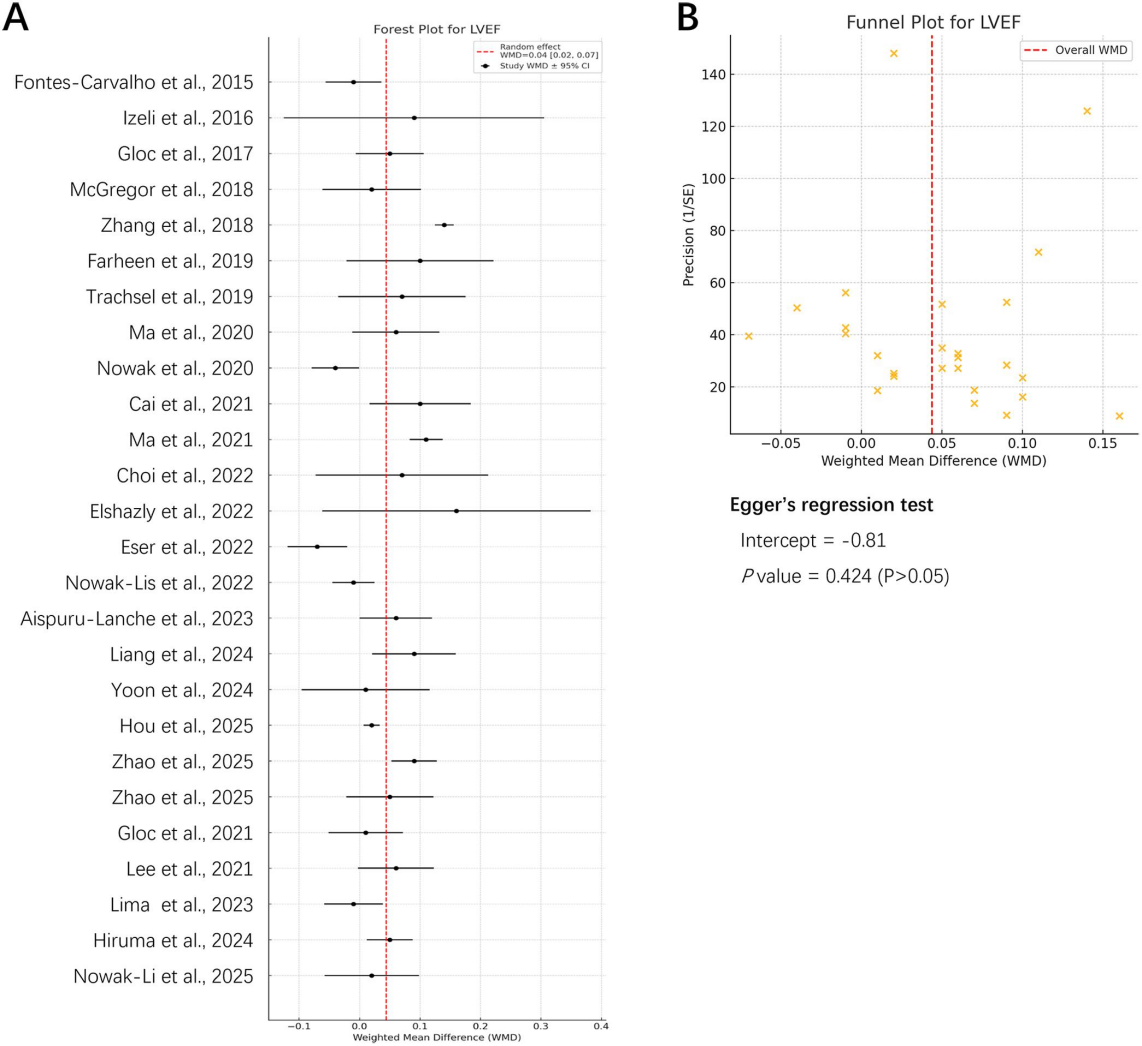
Meta-analysis and bias assessment of exercise intervention effects on LVEF (*n* = 26). **(A)** Forest plot of the effect of exercise intervention on LVEF in MI patients; **(B)** funnel plot of publication bias on the effect of exercise intervention on LVEF in MI patients.

LVEF serves as a key parameter for assessing left ventricular systolic function, reflecting the percentage of blood ejected per heartbeat (61). Extensive research indicates that LVEF, as a surrogate marker of myocardial contractility, is a significant predictor of morbidity and mortality in MI patients (61, 62). Consequently, the present study extracted 26 articles involving LVEF from the 42 included publications for meta-analysis and subgroup analysis. In order to ensure the reliability of subsequent results, a re-evaluation of bias risk was conducted for the 26 articles under consideration (Figure 4).

The results demonstrated that the 95% confidence intervals for effect sizes in most studies crossed zero, but the overall pooled effect was WMD = 0.048 [95% CI (0.020, 0.076)], indicating that exercise intervention significantly improved LVEF. The heterogeneity of the data was assessed using the *Tau²*, fully accounting for variability across studies and enhancing the reliability of the pooled effect.

Regarding publication bias, the funnel plot (Figure 4) showed symmetrical distribution on both sides of the red dashed line, indicating no apparent bias. The Egger regression test further revealed an intercept of −0.81 with a *P*-value of 0.424 (>0.05), failing to reach statistical significance. This finding indicates that there is no substantial small-sample effect or publication bias. The Egger regression results did not reject the null hypothesis, suggesting no significant publication bias, consistent with the symmetry observed in the funnel plot.

**Figure 5:**
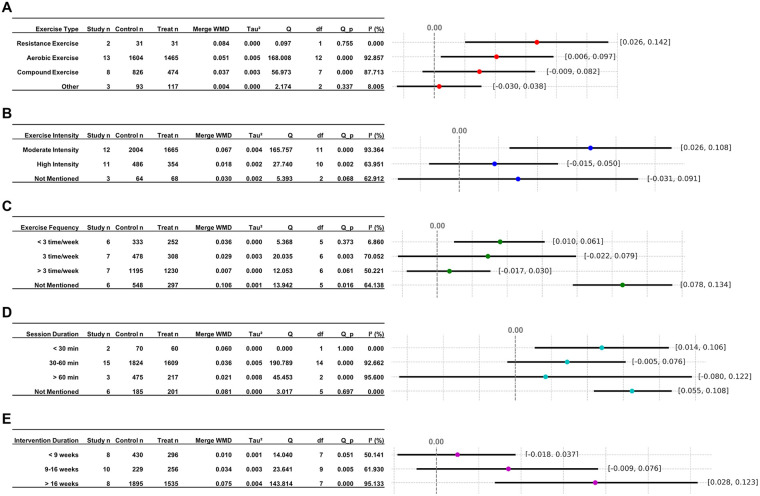
Subgroup analysis of LVEF based on different exercises. **(A)** Subgroup analysis of exercise type; **(B)** subgroup analysis of exercise intensity; **(C)** subgroup analysis of exercise frequency; **(D)** subgroup analysis of session duration; **(E)** subgroup analysis of intervention duration.

Subgroup analysis by exercise type was performed (Figure 5). The test for subgroup differences (interaction) indicated a statistically significant between-subgroup difference (*Q* = 16.29, *df* = 3, *P* = 0.001), suggesting that the effects on improving LVEF differed significantly across exercise modalities. RE demonstrated the largest effect size [WMD = 0.084, 95% CI (0.026, 0.142)], followed by AE [WMD = 0.051, 95% CI (0.006, 0.097)], followed by the CE group [WMD = 0.037, 95% CI (–0.009, 0.082)], and finally the other types (OT) group [WMD = 0.004, 95% CI (–0.030, 0.038)]. Heterogeneity analysis revealed varying degrees of inconsistency across the four groups regarding LVEF improvement (OT: *I²* = 8.005%; AE: *I²* = 92.857%; CE: *I²* = 87.713%; RE: *I²* = 0%), suggesting substantial differences in intrinsic LVEF enhancement between the AE and CE groups. It is noteworthy that RE group yielded the most substantial effect size with no observed heterogeneity. Nevertheless, the limited number of studies included, specifically only two, constrained the group's representativeness.

Subgroup analysis by exercise intensity was performed (Figure 5). The test for subgroup differences indicated a statistically significant between-subgroup difference (*Q* = 44.65, *df* = 2, *P* < 0.001), suggesting that the effects on improving LVEF differed significantly across exercise intensities. The pooled WMD was highest in the moderate intensity (MI) group [WMD = 0.067, 95% CI (0.026, 0.108)], followed by the not mentioned (NM) group [WMD = 0.030, 95% CI (−0.031, 0.091)], with the lowest effect size observed in the high-intensity (HI) group [WMD = 0.018, 95% CI (−0.015, 0.050)]. Heterogeneity analysis revealed substantial variability across the three groups regarding LVEF improvement (NM: *I²* = 62.912%; MI: *I²* = 93.364%; HI: *I²* = 63.951%), suggesting exercise intensity may influence LVEF enhancement. Collectively, the moderate-intensity group demonstrated superior intervention outcomes in comparison to the other intensity groups in the present study.

Subgroup analysis by exercise frequency was performed (Figure 5). The test for subgroup differences indicated a statistically significant between-subgroup difference (*Q* = 62.05, *df* = 3, *P* < 0.001), suggesting that the effects differed significantly across exercise frequency categories. The pooled WMD was highest in the NM group [WMD = 0.106, 95% CI (0.078, 0.134)], followed by the < 3 times/week group [WMD = 0.036, 95% CI (0.010, 0.061)], followed by the 3 times/week group [WMD = 0.029, 95% CI (−0.022, 0.079)], and finally the > 3 times/week group [WMD = 0.007, 95% CI (−0.017, 0.030)]. Heterogeneity analysis revealed varying degrees of heterogeneity across the four groups regarding LVEF improvement (NM: *I²* = 64.138%; < 3: *I²* = 6.860%; > 3: *I²* = 50.221%; = 3: *I²* = 70.052%), suggesting differential effects of exercise intervention frequency on LVEF enhancement. Furthermore, although the NM group exhibited the largest effect size, it was not representative. Consequently, the < 3 times/week group demonstrated superiority over other frequency groups in the studies.

Subgroup analysis by session duration was performed (Figure 5). The test for subgroup differences indicated a statistically significant between-subgroup difference (*Q* = 192.14, *df* = 3, *P* < 0.0001), suggesting that the effects differed significantly across session duration categories. The pooled WMD was highest in the NM group had the largest effect size [WMD = 0.081, 95% CI (0.055, 0.108)], followed by the < 30 min group [WMD = 0.060, 95% CI (0.014, 0.106)], then the 30–60 min group [WMD = 0.036, 95% CI (−0.005, 0.076)], and finally the > 60 min group [WMD = 0.021, 95% CI (−0.080, 0.122)]. Heterogeneity analysis revealed no significant heterogeneity between the < 30 min (*I²* = 0.000%) and NM groups (*I²* = 0.000%). However, the 30–60 min (*I²* = 92.662%) and > 60 min groups (*I²* = 95.600%) exhibited high heterogeneity in improving LVEF, suggesting that the session duration significantly influences the effect of exercise intervention on LVEF improvement. Despite the fact that the NM group demonstrated the most substantial effect size, it was not a representative sample. Consequently, the < 30 min group possesses a heightened reference value in comparison to other duration groups.

Subgroup analysis by intervention duration was performed (Figure 5). The test for subgroup differences indicated a statistically significant between-subgroup difference (*Q* = 62.05, *df* = 2, *P* < 0.001), suggesting that the effects differed significantly across intervention duration categories. The pooled WMD was highest in the > 16 weeks group [WMD = 0.075, 95% CI (0.028, 0.123)], followed by the 9–16 weeks group [WMD = 0.034, 95% CI (−0.009, 0.076)], while the < 9 weeks group had the smallest effect size [WMD = 0.010, 95% CI (−0.018, 0.037)]. Heterogeneity analysis revealed significant variability across all three groups regarding LVEF improvement (> 16W: *I²* = 95.133%; 9–16W: *I²* = 61.930%; < 9W: *I²* = 50.141%), suggesting that intervention duration may influence the efficacy of exercise interventions on LVEF. Collectively, the > 16 weeks group demonstrated superior LVEF improvement in comparison to other intervention durations.

### 3.6 Effects of exercise interventions on exercise performance in patients with MI

**Figure 6:**
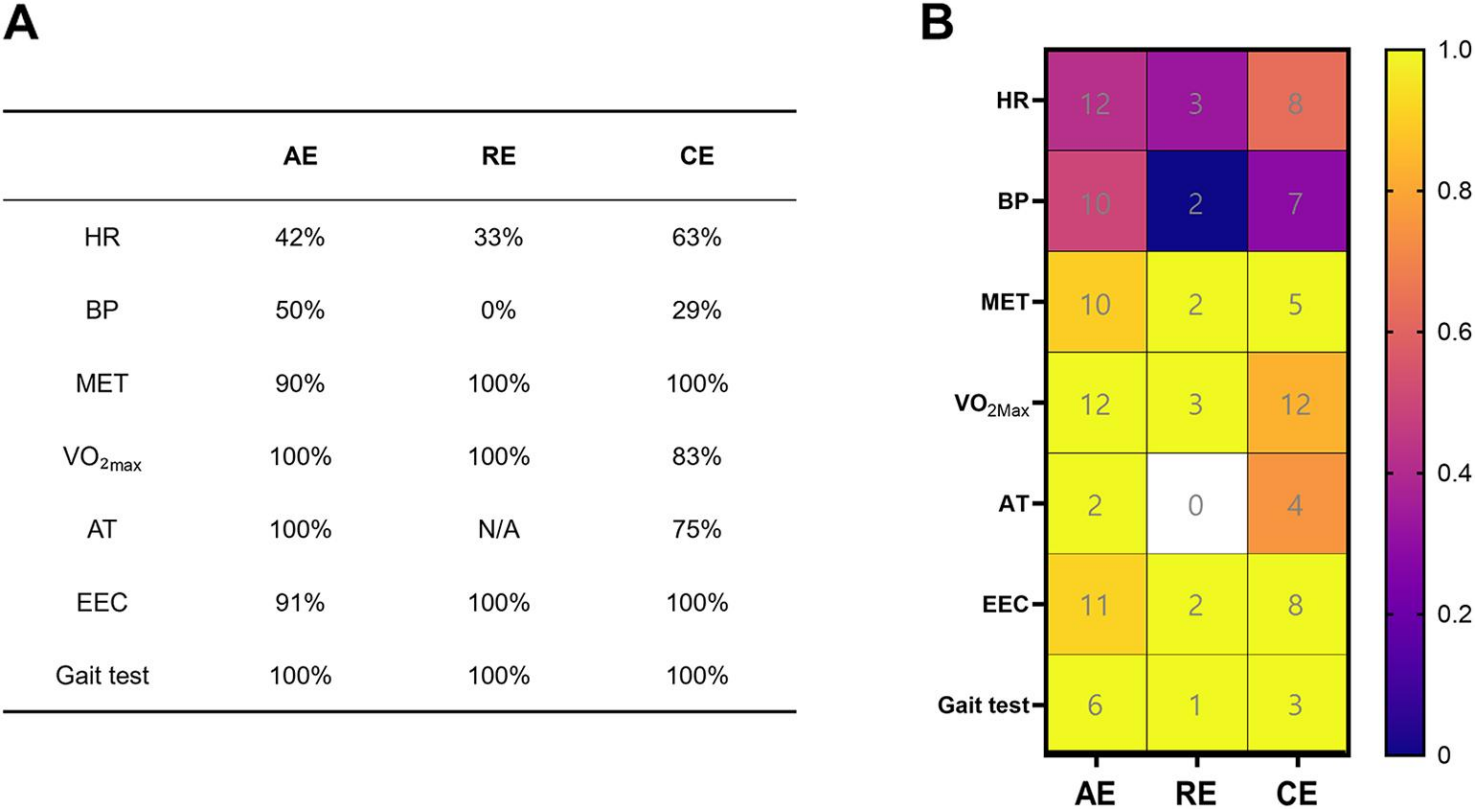
Research frequency and effectiveness distribution of performance indicators across different exercise types. **(A)** Different exercise types and their validity in assessing exercise performance in MI patients; **(B)** heatmap of exercise performance validity across different exercise types in MI patients; AE, aerobic exercise; RE, resistance exercise; CE, compound exercise; HR, heart rate; BP, blood pressure; MET, metabolic equivalent of task; VO_2_max, maximal oxygen uptake (mL/kg/min); AT, anaerobic threshold; EEC, endurance exercise capacity.

Among the 42 included studies, 39 investigated the effects of exercise interventions on the exercise performance of MI patients (Supplementary Table S3), employing visual heatmaps and validity statistical analysis (Figure 6). The heatmap illustrates the research frequency (numbers) and validity distribution (colors) of performance-related indicators across different exercise types. The frequency distribution reveals that maximal oxygen uptake (VO₂max), heart rate (HR), and endurance exercise capacity (EEC) indicators were studied more frequently than others, with total frequencies of 27 times (VO₂max; AE: *n* = 12; RE: *n* = 3; CE: *n* = 12), 23 times (HR; AE: *n* = 12; RE: *n* = 3; CE: *n* = 8), and 21 times (EEC; AE: *n* = 11; RE: *n* = 2; CE: *n* = 8), highlighting their central role in exercise performance. Blood pressure (BP; AE: *n* = 10; RE: *n* = 2; CE: *n* = 7), metabolic equivalent of task (MET; AE: *n* = 10; RE: *n* = 2; CE: *n* = 5), and Gait Tests (AE: *n* = 6; RE: *n* = 1; CE: *n* = 3) also received relatively high attention. In contrast, anaerobic threshold (AT; AE: *n* = 2; RE: *n* = 0; CE: *n* = 4) received the least attention, appearing in only a small number of studies and being entirely absent from RE. This may stem from its physiological significance and measurement methods not aligning with the research context, leading to its frequent omission in study designs.

In terms of color intensity, MET, EEC, VO₂max, and the Gait test demonstrated high validity across all forms of exercise types (with validity values ranging from 80% to 100%), indicating that these metrics possess broad consistency and representativeness in exercise intervention studies. AT, a crucial physiological indicator reflecting aerobic metabolism levels and exercise endurance, demonstrated relatively high validity in AE and CE. However, data concerning AT were not available in RE, which may be indicative of limited application or representativeness of this indicator in RE studies. Conversely, BP exhibited low validity across all exercise types, suggesting its applicability as an exercise performance metric may be limited. HR demonstrated the lowest validity in RE, indicating its application or stability in RE may be less effective than in AE and CE. In summary, MET, VO2max, EEC, and the Gait test are the most frequently studied and highly valid key indicators across different exercise types in the physical performance of MI patients. It is recommended that these be prioritized as key indicators in subsequent research.

### 3.7 Effects of exercise interventions on quality of life in patients with MI

Among the 42 included studies, eight investigated the effects of exercise interventions on the quality of life of MI patients (Table 3). These comprised 4 studies on AE, 3 on CE, and 1 on RE alone. Quality of life assessment tools employed in these studies included the SF-36 (23, 59), MacNew QLMI (30), SAQ (39, 40), CROQ (46), EQ-5D, KASI (52), and WHOQOL-BREF (54). Findings indicated that all 4 AE training significantly improved MI patients’ quality of life (*P* < 0.05), primarily in physical functioning, emotional well-being, social functioning, and mental health domains. Regarding RE training, studies demonstrated significant post-intervention improvements in MI patients’ quality of life (*P* < 0.05). However, among 3 studies CE training, only 2 demonstrated significant post-intervention improvements in MI patients’ quality of life (*P* < 0.05). The third study reported no significant post-intervention improvement in MI patients’ quality of life (EQ-5D, *P* = 0.332; KASI, *P* = 0.195).

**Table 3:** The impact of exercise intervention on quality of life in patients with MI (*n* = 8).

Author/Year	Type	Intervention duration	Frequency	Session duration	Intensity	Evaluation tool	Main results
Lee et al., 2017 (23)	AE	8W	3 t/w	60 min	High	SF-36	PF, RP, BP, GH, VT, SF, RE, MH, PCS, MCS (*P* < 0.05)
Ul-Haq et al., 2019 (30)	CE	8W	1 t/w	30 min	NM	MacNew QLMI	Quality of Life (*P* < 0.001)
Wang et al., 2021 (39)	CE	4W	3 t/w	50 min	Moderate	SAQ	Quality of Life (*P* = 0.042)
Bolatbekov et al., 2022 (40)	AE	24W	NM	NM	NM	SAQ	PL (*P* = 0.014) AS (*P* = 0.046) AF (*P* = 0.150) TS (*P* = 0.014) DP (*P* = 0.007)
Qu et al., 2022 (46)	AE	3W	NM	NM	NM	CROQ	Symptom scoring; physical, cognitive and psychosocial functioning (*P* < 0.01)
Jo et al., 2024 (52)	CE	6W	3 t/w	45–50 min	Moderate	EQ-5D KASI	EQ-5D (*P* = 0.332) KASI (*P* = 0.195)
Liang et al., 2024 (54)	RE	12W	NM	NM	NM	WHOQOL-BREF	Quality of Life (*P* < 0.05)
Zhao et al., 2025 (59)	AE	48W	NM	NM	Moderate	SF-36	PF, RP, BP, GH, VT, SF, RE, MH (*P* < 0.001)

SF-36, 36-item short form health survey; MacNew QLMI, MacNew quality of life after myocardial infarction questionnaire; SAQ, Seattle angina questionnaire; EQ-5D, EuroQoL-5 dimensions; KASI, Korean activity scale/index; WHOQOL-BREF, World Health Organization quality of life—brief version; PF, physical functioning; RP, role-physical; BP, bodily pain; GH, general health; VT, vitality; SF, social functioning; RE, role-emotional; MH, mental health; PCS, physical component summary; MCS, mental component summary; PL, physical limitation; AS, angina stability; AF, angina frequency; TS, treatment satisfaction; DP, disease perception; NM, not mentioned; t/w, times/week.

The foregoing findings indicate that diverse exercise interventions exert a positive influence on the quality of life of MI patients. However, due to considerable variation in exercise type, intensity, frequency, session duration, intervention duration and assessment tools employed, certain studies have not explicitly specified the intensity, frequency, or duration of interventions. These factors have the potential to impact on the consistency and interpretation of results.

### 3.8 Changes in the incidence of adverse cardiovascular events

Evidence from multiple studies indicates that AE or CE can significantly reduce the incidence of adverse cardiovascular events in patients with MI and improve overall health outcomes.

With respect to AE, Cai et al. (33), Zhang et al. (27), and Hou et al. (57) observed that, following interventions lasting 3, 24, and 48 weeks, respectively, AE significantly reduced the risks of angina pectoris, rehospitalization rate, heart failure, arrhythmia, and cardiac mortality in patients with MI. One study further confirmed that AE interventions-maintained reductions in the incidence of adverse cardiovascular events over a 30-month follow-up period (51). Moreover, Lee et al. (37) demonstrated that cardiac rehabilitation programs centered on AE were significantly associated with a lower risk of adverse cardiovascular events, thereby establishing AE as a key determinant of cardiovascular outcomes.

With respect to CE, Wang et al. (39) and Ma et al. (38) observed that, following 4- and 24-week interventions, respectively, this approach significantly reduced the incidence of adverse cardiovascular events in patients with MI while concomitantly improving their quality of life. Fontes-Carvalho et al. (19) reported that no adverse cardiovascular events occurred during the 8-week aerobic–resistance program or immediately thereafter; however, during the two-year follow-up, three non-fatal MIs were observed in the exercise group, whereas one cardiac death occurred in the control group. Hiruma et al. (50) further demonstrated that CE reduced the incidence of adverse cardiovascular events in MI patients, independent of baseline exercise capacity.

In summary, the present study demonstrates that both AE and CE exhibit consistent cardiovascular protective effects in MI patients. These interventions have been shown to reduce the risk of adverse events while concomitantly promoting long-term rehabilitation and health maintenance.

## 4 Discussion

### 4.1 Exercise prescription selection for the rehabilitation phase of MI patients

Considering the findings, it is recommended that exercise prescriptions for MI patients should primarily consist of moderate-intensity AE or RE. Exercise intensity is recommended to be controlled at rating of perceived exertion levels 11–13 (resistance) or 40%–60% of maximum heart rate (aerobic), with single sessions lasting < 30 min, ≤2 times weekly. It is imperative that the intervention duration is sustained for a period exceeding 16 weeks to ensure rehabilitation efficacy. Gradually extend session duration and increase training frequency as tolerated, promoting sustained optimization of cardiopulmonary function and steady rehabilitation progress while ensuring cardiac workload safety. It is imperative that exercise prescription adheres to the principles of individualization, progression, and safety first, with adjustments tailored to the patient's age, baseline cardiac function, comorbidities, and exercise tolerance. During exercise sessions, professional supervisors must continuously monitor the patient's physical condition. In the event of the onset of symptoms such as chest tightness, chest pain, palpitations, or dyspnoea occur, it is imperative that exercise is immediately halted and appropriate intervention administered. This approach is designed to ensure that rehabilitation exercises are conducted in a manner that is both safe and effective, thereby maximising the recovery outcomes for patients.

### 4.2 Avoiding and balancing common exercise risks during the rehabilitation period for MI patients

Notwithstanding the considerable body of research indicating that exercise interventions exert a markedly positive effect on cardiac function recovery and exercise performance improvement in MI patients, it is important to note that exercise can have deleterious effects. Its therapeutic efficacy is highly contingent upon rigorous individual risk assessment and the scientific formulation of exercise prescriptions. Consequently, when prescribing exercise regimens for MI patients, it is imperative to conduct a comprehensive evaluation of potential risk factors during rehabilitation to ensure both the safety and efficacy of the exercise intervention.

The following risk factors for exercise are well-documented: arrhythmia, heart failure, angina or recurrent MI, blood pressure fluctuations, syncope (63, 64), and the presence of other chronic conditions (e.g., diabetes mellitus, hypertension, obesity) (65). Furthermore, psychological factors (anxiety, depression, fear) (66), medication factors (e.g., statins, *β*-blockers) (67), inappropriate exercise intensity or type, and external factors (e.g., lack of professional supervision or monitoring equipment) can all impact patients’ exercise adherence and cardiovascular responses. Inadequate assessment and management of these risk factors during exercise can result in lead to severe complications or even life-threatening events. Consequently, the rational avoidance and management of these exercise-related risk factors are crucial for ensuring the safety of rehabilitation exercise in MI patients.

For example: (1) Arrhythmia: Pre-exercise screening via electrocardiogram (ECG) and exercise stress testing is essential. High-risk patients should exercise under ECG monitoring. Immediate cessation of exercise and prompt intervention are required upon detection of significant arrhythmia. (2) Heart failure: Exercise prescriptions should be individually tailored based on the patient's ejection fraction and symptom grading, avoiding high-intensity or excessive exertion. (3) Angina or recurrent infarction: Exercise intensity must be strictly controlled, adhering to the principle of gradual progression to avoid exceeding the patient's safe tolerance range. Exercise should cease immediately, and appropriate intervention initiated upon the onset of chest pain. (4) Blood pressure fluctuations: Dynamically monitor blood pressure during exercise, avoiding activity when readings are excessively high or low. (5) Syncope: Be vigilant for syncope caused by orthostatic hypotension, arrhythmia, or excessive exercise fatigue. Intervention sites must have emergency protocols in place. (6) Coexisting chronic conditions: Diabetes mellitus: Monitor blood glucose before and after exercise; avoid exercising on an empty stomach or during peak insulin action. Carry sugar tablets to prevent hypoglycemia. Hypertension: Ensure blood pressure is within safe limits (generally <160/100 mmHg) prior to exercise (68). Closely monitor blood pressure during exercise; avoid excessive breath-holding and explosive movements. Obesity: Employ low-impact aerobics activities (e.g., walking, cycling, swimming) to minimize joint injury risk. Gradually increase intensity to prevent sudden cardiac overload (7). Psychological factors: Utilize screening scales and interviews to understand patients’ perceptions of their condition, identifying potential psychological issues. Combine health education with progressive exercise prescriptions to alleviate negative emotions and gradually build confidence. Where necessary, psychological counselling or pharmacological interventions may be integrated to alleviate negative emotions, enhance exercise adherence, and ensure sustained and effective rehabilitation outcomes (8). Pharmacological Factors: Clinical follow-ups and regular patient reviews enable timely detection of early medication-related adverse reactions. Adjustments to dosage or treatment regimens should be made as appropriate to minimize the detrimental impact of side effects on exercise rehabilitation (9). Inappropriate exercise intensity, type, and external factors: Develop individualized, progressive exercise prescriptions based on cardiac function classification and exercise test results to avoid overload. Exercise should ideally be conducted in supervised rehabilitation centers equipped with monitoring devices to promptly detect abnormalities. Where professional supervision is unavailable, intensified patient education and home guidance are required to enhance self-monitoring capabilities and ensure rehabilitation safety.

Whilst risk mitigation is paramount, it is equally crucial to assess cardiovascular risk through meticulous methods such as cardiopulmonary exercise testing (CPET), echocardiography, and Holter monitoring (68, 69) to determine whether patients have exercise contraindications or require specialized supervision. Concurrently, attention should be paid to the patient's physical fitness level, exercise tolerance, and prior exercise history to rationally determine exercise intensity and modality. Therefore, when formulating exercise prescriptions, the fundamental principles of “FITT-VP” should be adhered to after thorough risk factor assessment and control (70). These prescriptions must be dynamically adjusted according to the patient's actual condition to ensure both the scientific validity and safety of the intervention.

### 4.3 Limitations of the current study

(1) Approximately 41% of the studies are non-RCTs and have a high risk of bias, which may impact the accuracy and reliability of the research findings.
(2) Due to differences in outcomes and measurement indicators across some studies, a meta-regression analysis could not be performed, which limits the comprehensiveness and detailed analysis of the results.
(3) The use of LVEF as the sole indicator for cardiac function assessment may have limitations regarding regional cardiac function metrics.
(4) There is considerable heterogeneity in the intervention methods at the research centers (e.g., type, session duration, frequency, intensity, intervention duration), which may impact the results and limit the generalizability of the study conclusions.
(5) A substantial number of studies focused primarily on moderate-intensity endurance exercise, with insufficient attention to other exercise types, intensity, and session duration. This gap in knowledge may limit insights into the effects of exercise prescription. Notably, low-to-moderate intensity RE has also demonstrated highly significant modulatory effects on cardiac function and exercise performance indicators, yet research in this area remains scarce (only 2 studies). This research gap may consequently impact the development and implementation of exercise prescriptions. In addition, some studies did not report key prescription components—such as intensity, frequency, and session duration (NM). These NM studies yielded the largest pooled effects in the meta-analysis; however, incomplete reporting may lead to misclassification and increased heterogeneity, thereby reducing the reliability of the overall effect estimates. Therefore, NM studies were excluded from the primary analyses due to insufficient prescription details for reliable classification and were summarized descriptively.

### 4.4 Future research directions

In order to address the limitations of the studies, future research can be improved in the following areas: (1) Related studies should strive for greater consistency in the design and measurement methods of outcome indicators to facilitate more in-depth subgroup or regression analyses in subsequent research. In addition, long-term follow-up of ≥2 years is recommended to evaluate the sustainability of intervention effects and changes in clinical outcomes, thereby facilitating more in-depth subgroup or regression analyses in subsequent research. (2) Methodological rigor should be strengthened to minimize the risk of bias, with particular emphasis on quality control in the randomization process, blinding procedures, allocation concealment, and outcome reporting. Meanwhile, priority should be given to head-to-head RCT that directly compare key exercise prescription parameters (e.g., modality, intensity, frequency, session duration, and intervention duration) to generate more robust and clinically informative evidence. (3) Subsequent studies may incorporate multidimensional indicators beyond LVEF, such as diastolic function and right ventricular function. The utilization of unified measurement standards and integrated evaluation systems has the potential to enhance the accuracy and comprehensiveness of cardiac function assessment. (4) Standardization and stratified design of intervention protocols may help reduce the impact of heterogeneity and support the exploration of individualized, precision-oriented intervention pathways. In addition, expanding sample size and broadening recruitment sources may enhance the generalizability and representativeness of the evidence. (5) Future research should examine exercise interventions of different modalities, intensities, and durations, with particular attention to expanding evidence on low-to-moderate intensity resistance training. Direct comparisons between supervised and home-based programs are also needed. Furthermore, cost-effectiveness analyses should be incorporated to provide a more comprehensive basis for the scientific development, implementation, and resource allocation of exercise prescriptions.

## 5 Conclusion

This systematic review and meta-analysis demonstrate that exercise interventions are significantly associated with improvements in cardiac function, exercise performance, and quality of life among MI patients and are also linked to lower rates of adverse cardiovascular events. Regarding exercise variables, moderate-intensity AE or RE with a session duration of <30 min, a frequency of <3 sessions per week, and an intervention duration of >16 weeks was associated with greater improvements in outcomes. However, due to considerable heterogeneity among studies and the limited number of studies on RE, interpretation of these associations requires caution, and the influence of potential confounding factors cannot be ruled out. Future research necessitates more high-quality, rigorously designed studies, particularly those further evaluating the strength and stability of the association between RE and MI rehabilitation outcomes, to provide more reliable evidence-based guidance for clinical practice.

## Data Availability

The original contributions presented in the study are included in the article/Supplementary Material, further inquiries can be directed to the corresponding authors.
