# Supplementary material for: Effectiveness and selection of exercise prescriptions for myocardial infarction patients: a systematic review and meta-analysis
Source: Front Cardiovasc Med. 2026 Feb 23;13:1739046. doi: 10.3389/fcvm.2026.1739046 (PMC12968203; doi:10.3389/fcvm.2026.1739046)
Supplement: Supplementary file 1 [file Datasheet1.docx]

**Supplementary Material**

**Table S1. Basic characteristics of included studies (n=42)**

| **Author/Year** | **Research design** | **Sample size** | **Age** | **Exercise type** | **Intensity** | **Frequency, Session / Intervention duration** | **Progression** | **Control intervention** | **Outcome** |
| --- | --- | --- | --- | --- | --- | --- | --- | --- | --- |
| Fontes-Carvalho et al., 2015 | RCT | CG:89  IG:86 | CG:55.4±10.3  IG:55.9±10.8 | Aerobic exercise + resistance training | 70–85% HRmax | 3 t/w  70min  8W | Endurance intensity progressed (weeks 1–4: cycling; weeks 5–8: treadmill); resistance load progressed from 2 kg×10 reps. | usual care | LVEF; VO₂peak; Exercise duration; MACE |
| Omiya et al., 2015 | Quasi-experimental study | CG:24  IG1:23  IG2:23 | CG:57.3±12.5  IG1:61.8±6.9  IG2:61.2±9.8 | IG1: Aerobic exercise + resistance training  IG2: Aerobic exercise + resistance training | RPE11-13 | 2 t/w  60min  12W | NM | Aerobic exercise + resistance training | VO₂peak; HRpeak |
| Izeli et al., 2016 | Quasi-experimental study | CG:8  IG:18 | CG:50.3±9.7  IG:54.1±7.0 | Aerobic exercise | 50-70%HRR | 4 t/w  40min  12W | NM | usual care | LVEF; VO₂peak; MET; HRpeak; SBPpeak; Speed |
| Gloc et al., 2017 | RCT | CG:10  IG1:20  IG2:20 | CG:55.40±8.25  IG1:57.50±9.51  IG2:56.40±9.85 | IG1: Indoor cycling course  IG2: Bicycle training+resistance training+aerobics | IG1:50-80% HRR; RPE 14  IG2:50-80% HRR; RPE 14 | 5 t/w  30min  4W | NM | usual care | LVEF |
| Lee et al., 2017 | Quasi-experimental study | CG:50  IG:72 | CG:54.3±7.7  IG:54.8±8.3 | Aerobic exercise | 40-80%HRR | 3 t/w  60min  8W | NM | usual care | VO₂max; MET; HRmax; ETmax; SF-36 |
| Santi et al., 2018 | RCT | CG:10  IG1:10  IG2:10 | Average54.8±8.3 | IG1: Intermittent aerobic exercise  IG2: Aerobic exercise | IG1:85-95% HR  IG2:60-70% HR | 3 t/w  40min  12W | NM | NM | VO₂peak; HRpeak; SBP |
| Kunjan et al., 2018 | Quasi-experimental study | CG:43  IG:40 | CG:56.8±11.1  IG:54.2±10.7 | Aerobic exercise | RPE 5-8 | NM  NM  12W | NM | usual care | MVPA;6MWT |
| McGregor et al., 2018 | Quasi-experimental study | CG:16  IG:15 | CG:54.1±10.6  IG:57.0±10.7 | Aerobic exercise + resistance training | 60-80% VO2 peak | 2 t/w  25-40min  10W | NM | usual care | LVEF; VO₂peak; VT; HRrest; SBP; Wmax |
| Zhang et al., 2018 | RCT | CG:65  IG:65 | CG:69.8±10.4  IG:70.3±10.7 | Aerobic exercise | RPE 11-16;60-75% HRmax | 2-5 t/w  15-45min  24W | Phase II (from week 2 post-discharge) → Phase III (from month 3 to month 6). Training load was progressed across phases. Phase II: HR <130 bpm or ≤resting HR +30 bpm; RPE ≤11–15; 50 → 250–300 kcal·h⁻¹. Phase III: 60–75% HRmax; RPE ≤12–16; 300–400 kcal·h⁻¹; volume increased to 30–45 min, 3–5 sessions/week. Training was stopped or the load was reduced in case of discomfort. | usual care | LVEF; exercise time; 6MWT; MACE |
| Farheen et al., 2019 | RCT | CG:13  IG:13 | CG:69.8±10.4  IG:70.3±10.7 | Aerobic exercise + resistance training | 65%-85% THR;30-50% 1RM | 3 t/w  NM  6W | NM | Intermittent aerobic training | LVEF |
| Trachsel et al., 2019 | RCT | CG:10  IG:9 | CG:57±13  IG:60±10 | Resistance Training | RPE 15 | 2 t/w  50-60min  12W | NM | usual care | LVEF; VO₂peak; HRpeak; SBPpeak; Peak Workload (W) |
| Ul-Haq et al., 2019 | RCT | CG:96  IG:99 | Average53±8.3 | Aerobic exercise + resistance training | NM | 1 t/w  30min  8W | NM | usual care | MacNew QLMI |
| Ma et al., 2020 | Retrospective observational study | CG:46  IG:32 | CG:57.6±8.5  IG:59.3±7.2 | Walking training | 50%-70% HRR | 3 t/w  20min  24W | NM | usual care | LVEF、6MWT |
| Nowak et al., 2020 | RCT | CG:20  IG:24 | CG:57.45±8.12  IG:60.92±7.8 | Aerobic exercise + resistance training | 60-80% HRR; RPE 12-14 | 5 t/w  90min  4W | Intensity was prescribed using training heart rate: starting at 60% HRR and increased by 10% HRR after every 5 sessions until 80% HRR (≈ Borg RPE 14). | Aerobic exercise + resistance training | LVEF; VO₂max; MET; HRrest; SBPrest; Exercise distance |
| Cai et al., 2021 | Retrospective cohort study | CG1:122  CG2:32  IG1:56  IG2:22 | CG1:61.20±11.31  CG2:57.03±6.7  IG1:58.84±10.37  IG2:57.09±9.17 | Cycling training | RPE 11-13 | 3-4 t/w  20min  192W | Early in-hospital CR started ~48 h after PCI and lasted 2 weeks (3 supervised interval cycling sessions/week + 4 electrical stimulation sessions/week). CPX before discharge guided a home program (walking/cycling 3–4×/week at VT heart rate) with 3-monthly follow-up (~4 years), but home adherence data were not recorded. | usual care | MACE |
| Cai et al., 2021 | RCT | CG:30  IG:30 | CG:58±8  IG:55±9 | Walking training | 70-80% HRmax | NM  NM  24W | NM | usual care | LVEF; VO₂peak; MET; AT; exercise time |
| Dehghani et al., 2021 | RCT | CG:40  IG:40 | CG:53.25±7.15  IG:51.95±7.27 | Walking training | RPE 11-13 | 5 t/w  50min  8W | From **weeks 2–8**, daily steps recorded by a **pedometer** were increased by **10% per week** (baseline ≈ **3,500 steps/day**, determined in a pilot test). | usual care | HRmax;SBP rest |
| Gloc et al., 2021 | RCT | CG:32  IG:32 | CG:55.31±6.45  IG:53.4±4.31 | Cycling training | 60-80% HRR | 5 t/w  30min  4W | NM | Aerobic exercise + resistance training | LVEF; VO₂max; MET; HR rest; SBP rest; Test duration |
| Lee et al., 2021 | Quasi-experimental study | CG:225  IG1:63  IG2:117 | CG:65.2±12.4  IG1:61.4±9.5  IG2:62.9±11.2 | Aerobic exercise | 60-85% HRmax | 3 t/w  50min  6W | arget heart rate was determined using the **Karvonen formula** and progressed over 6 weeks: **weeks 1–2, 60% HRmax; weeks 3–4, 70% HRmax; weeks 5–6, 85% HRmax**. | usual care | LVEF; MACE |
| Ma et al., 2021 | Quasi-experimental study | CG:369  IG:104 | CG:65.6±10.752  IG:50.39±9.381 | Aerobic exercise + resistance training | 50%-60% HRmax | NM  40-110min  24W | NM | usual care | LVEF; MACE; 6-MWD |
| Wang et al., 2021 | RCT | CG:90  IG:91 | CG:62.2±9.5  IG:61.4±9.1 | Aerobic exercise + resistance training | RPE 9-13 | 3 t/w  50min  4W | NM | usual care | 6MWT; SAQ; MACE |
| Bolatbekov et al., 2022 | Quasi-experimental study | CG:32  IG:86 | CG:57±8.9  IG:57±8.5 | Aerobic exercise | NM | NM  NM  24W | An individualized daily training program was implemented, starting with a low workload and progressively increasing intensity. Progression was based on a combined assessment of **heart rate response** and **Borg RPE (0–10)**; target HR was calculated using the **Karvonen formula**. HR was continuously monitored by **ECG** during training, and Borg ratings were recorded. | Aerobic exercise | VO₂max; MET; duration of training in seconds; 6MWT; SAQ |
| Choi et al., 2022 | Quasi-experimental study | CG:16  IG:26 | CG:64.3±8.70  IG:61.3±6.52 | Aerobic exercise | 40-85% HRR | 1-2 t/w  60min  12W | NM | usual care | LVEF; VO₂peak; MET; HRmax; SBPmax; exercise time |
| Elshazly et al., 2022 | RCT | CG:16  IG:16 | CG:51.1±9.6  IG:50.1±8.6 | Aerobic exercise | 40-60% HRR; RPE 11-13 | 3 t/w  35min  12W | NM | usual care | LVEF; MET; HRrest; DBP rest |
| Eser et al., 2022 | RCT | CG:34  IG:35 | CG:59（51-62）  IG:55（50-66） | HIIT | HR peak 90-95%; RPE ≥15 | 3 t/w  38min  9W | NM | MICT | LVEF; VO₂peak; HR; DBP; exercise time |
| Kambic et al., 2022 | RCT | CG:19  IG1:19  IG2:21 | CG:61（9）  IG1:61（7）  IG2:62（8） | IG1: Aerobic exercise + resistance training  IG2: Aerobic exercise + resistance training | IG1:50-80% PPO;35-40% 1RM  IG2:50-80% PPO;70-80% 1RM | 3 t/w  60min  12W | Resistance training intensity was progressed by %1-RM: **IG2, 70% → 80% 1-RM; IG1, 35% → 40% 1-RM.** At **week 8 (session 22), 1-RM was re-assessed,** and the prescription was recalculated based on the updated 1-RM, with further progression during the final 4 weeks. | Aerobic exercise | Arm curl test; 6MWT |
| Nowak-Lis et al., 2022 | RCT | CG:27  IG:36 | CG:55.19±8.03  IG:53.71±7.13 | Vibration training | 40 Hz, 2 mm amplitude (moderate intensity) | 5 t/w  30min  4W | NM | Aerobic exercise + resistance training | LVEF; VO₂max; MET; HR rest; SBP rest; Exercise duration |
| Qu et al., 2022 | Quasi-experimental study | CG:52  IG:55 | CG:56.21±9.77  IG:56.27±9.44 | Aerobic exercise | NM | NM  NM  3W | NM | usual care | CROQ |
| Aispuru-Lanche et al., 2023 | RCT | CG:24  IG1:28  IG2:28 | CG:57.0±7.2  IG1:59.0±9.6  IG2:58.9±8.0 | IG1: Low-volume HIIT  IG2: High-volume HIIT | IG1:85-95% HRR  IG1:85-95% HRR | 2 t/w  20min  16W | IG2: 20→40 min (progressive); IG1: NR (progression not reported). | Aerobic exercise | LVEF |
| Lima et al., 2023 | RCT | CG:27  IG:26 | CG:59±11  IG:60±9 | Aerobic exercise + resistance training | NM | 2 t/w  60min  16W | NM | usual care | LVEF; VO₂peak; AT; Basal HR; SBP Exercise duration; Exercise duration |
| Aispuru-Lanche et al., 2024 | RCT | CG:24  IG1:28  IG2:28 | CG:57.0±7.2  IG1:59.0±9.6  IG2:58.9±8.0 | IG1: Low-volume HIIT training  IG2: High-volume HIIT training | IG1:85-95% HRR  IG1:85-95% HRR | 2 t/w  20-40min  16W | NM | Aerobic exercise | HR rest; SBP rest |
| Hiruma et al., 2024 | Observational cohort study | CG:180  IG1:241  IG2:147 | CG:68.0±13.3  IG1:63.9±13.3  IG2:68.1±11.2 | Aerobic exercise + resistance training | RPE 13 | 1-3 t/w  50min  21W | NM | usual care | LVEF; VO₂peak; AT; HR rest; SBP rest; MACE |
| Hou et al., 2024 | Quasi-experimental study | CG:1247  IG:286 | CG:55.0（48.0,63）  IG:62.0（53.0,69.0） | Aerobic exercise | RPE 13-15 | 3-5 t/w  30min  12W | Over the 3-month intervention, intensity was targeted at **Borg RPE 13–15**, and training load and/or volume was **progressively increased** based on **DASI** assessment results. | usual care | VO₂peak; MACE |
| Jo et al., 2024 | RCT | CG:24  IG:24 | CG:56.0±7.0  IG:58.0±12.4 | Aerobic exercise + resistance training | RPE 11-13 | 3 t/w  45-50min  6W | NM | usual care | VO₂max; MET; HR rest; SBP rest; EQ-5D; KASI |
| Ksela et al., 2024 | RCT | CG:14  IG1:11  IG2:20 | CG:66.5（64.0-71.5）  IG1:65.0（62.5-68.0）  IG2:66.0（62.0-70.5） | Aerobic exercise + resistance training | 60-80% HRpeak | 12 t/w  30min  2W | NM | usual care | VO₂peak |
| Liang et al., 2024 | Quasi-experimental study | CG:21  IG1:18  IG2:19  IG3:21  IG4:22  IG5:26 | CG:53.08±11.80  IG1:54.61±9.37  IG2:58.11±12.81  IG3:55.42±12.54  IG4:53.61±10.22  IG5:55.07±13.67 | IG1: Continuous resistance training  IG2: Continuous aerobic exercise  IG3: Intermittent aerobic exercise  IG4: Interval resistance training  IG5: Inspiratory muscle training | NM | NM  NM  12W | NM | usual care | LVEF; VO₂peak; MET; HR peak; 6MWT; WHOQOL-BREF |
| Mitropouloset al., 2024 | RCT | CG:15  IG:15 | CG:53.5±6.6  IG:54.1±8.5 | Aerobic exercise, resistance training, and balance and flexibility training | VT1(moderate intensity), PRE 13-15 | 3 t/w  60min  26W | Aerobic training progression was primarily achieved by **adjusting intensity** based on **RPE responses** in conjunction with **recorded heart rate**. Resistance training progression was **RPE-triggered**: when **RPE was below the lower target limit (<13) for three consecutive sessions**, intensity was progressively increased by **increasing the distance from the resistance band (i.e., greater band stretch)** and/or **switching to a higher-resistance band**. | Aerobic exercise, resistance training, and balance and flexibility training | VO₂peak; Exercise duration |
| Yoon et al., 2024 | Quasi-experimental study | CG:8  IG:21 | CG:62（51,70）  IG:59（51,70） | Aerobic exercise + resistance training + flexibility Training + balance training | RPE 11-13 | NM  NM  24W | Exercise **intensity and duration** **gradually increased** and adjusted according to the prescribed program and patient responses. The prescription was guided by **risk stratification based on an exercise stress test**. After completion of outpatient rehabilitation, a **home-based cardiac rehabilitation (CR) prescription** was provided, reflecting **phase-based progression**. | usual care | LVEF |
| Hou et al., 2025 | Quasi-experimental study | CG:1081  IG:1081 | CG:60.8±10.4  IG:60.6±10.7 | Aerobic exercise | Inpatient: +10 to +20 bpm; post-discharge: +20–30 bpm (low-to-moderate intensity) | 3-5 t/w  30min  48W | Progression followed a target heart rate–based approach during the first month, with subsequent intensity adjustments guided by CPET re-assessments at 1, 3, 6, and 12 months. In case of discomfort, a step-down recovery strategy was applied: exercise was resumed only after complete symptom resolution, at an intensity below the level that provoked symptoms. | usual care | LVEF; VO₂; HR; SBP; MACE |
| Nowak-Li et al., 2025 | RCT | CG:17  IG:19 | CG:59.88±5.87  IG:58.52±6.09 | Aerobic exercise | NM | 5 t/w  40min  3W | **Workload:** Starting at **20 W**, increased by **5 W every 3 days**. | Aerobic exercise | LVEF; MET; HR peak; SBP peak; Test duration |
| Zhao et al., 2025 | RCT | CG:55  IG:55 | CG:59.52±8.78  IG:58.69±9.46 | Aerobic exercise | RPE 12-14 | NM  NM  48W | NM | usual care | LVEF; VO₂ max; MET; AT; SF-36 |
| Zhao et al., 2025 | RCT | CG:58  IG:60 | CG:60.1±11.4  IG:54.2±10.4 | Aerobic exercise + resistance training + flexibility training + passive joint movements + inspiratory muscle training + rehabilitation exercises+ balance training | 40-60% HRR | 3 t/w  NM  12W | **Phase 1:** Began with **passive joint movements** (toe flexion, ankle dorsiflexion) and, as tolerated, progressed to **activities of daily living**, with gradual increases in the **content and complexity** of training (muscle strengthening, rehabilitation exercises, resistance/balance/endurance training, and interval aerobic exercise). **Phase 2:** Transitioned to a **structured prescription: 4 consecutive weeks**, **3 sessions/week** of **moderate-intensity** training, including **aerobic exercise at 40–60% HRR, resistance training,** and **flexibility training**. | usual care | LVEF; VO_2_peak; MET; AT |

**CG**: Control group; **IG**: Intervention group; **t/w**: Times/Week; **W:** Week; **NM:** Not mentioned; **LVEF:** Left ventricular ejection fraction; **VO₂ peak:** Peak oxygen uptake; **VO₂ max:** Maximal oxygen uptake; **MET:** Metabolic equivalent of task; **AT:** Anaerobic threshold; **VT:** Ventilatory threshold; **HR peak:** Peak heart rate; **HRmax:** Maximum heart rate; **HR:** Heart rate; **RHR:** Resting heart rate; **BP:** Blood pressure; **Peak SBP:** Peak systolic blood pressure; **SBP:** Systolic blood pressure; **SBP rest:** Resting systolic blood pressure; **DBP rest:** Resting diastolic blood pressure; **DBP:** Diastolic blood pressure; **ET max:** Maximal exercise time; **MVPA:** Moderate to vigorous physical activity; **Wmax:** Maximum workload; **6MWT:** 6-Minute walk test; **6MWD:** 6-Minute walk distance; **MACE:** Major adverse cardiovascular events; **SF-36:** 36-item short form health survey; **MacNew QLMI:** MacNew quality of life after myocardial infarction questionnaire; **SAQ:** Seattle angina questionnaire; **EQ-5D**: EuroQoL-5 dimensions; **KASI:** Korean activity scale/index; **WHOQOL-BREF:** World health organization quality of life – brief version; **PF:** Physical functioning; **RP:** Role-physical; **BP:** Bodily pain; **GH:** General health; **VT:** Vitality; **SF:** Social functioning; **RE:** Role-emotional; **MH:** Mental health; **PCS:** Physical component summary; **MCS:** Mental component summary; **PL:** Physical limitation; **AS:** Angina stability; **AF:** Angina frequency; **TS:** Treatment satisfaction; **DP:** Disease perception.

**Table S2: GRADE quality of evidence for LVEF (n=26)**

| **Intervention** | **Comparation** | **Outcomes** | **Included MA** | **Risk of bias** | **Inconsistency** | **Indirectness** | **Imprecision** | **Other** | **Quality** |
| --- | --- | --- | --- | --- | --- | --- | --- | --- | --- |
| Aerobic exercise + resistance training | Usual care | LVEF | Fontes-Carvalho et al.,2015 | Serious | Not serious | Not serious | Not serious | Not assessable | Moderate |
| Indoor cycling course | Usual care | LVEF | Gloc et al.,2017 | Very serious | Not serious | Not serious | Serious | Not assessable | Very low |
| Aerobic exercise | Usual care | LVEF | Zhang et al.,2018 | Very serious | Not serious | Not serious | Not serious | Not assessable | Low |
| Aerobic exercise + resistance training | Intermittent aerobic training | LVEF | Farheen et al.,2019 | Very serious | Not serious | **Not serious** | Serious | Not assessable | Very low |
| Resistance Training | usual care | LVEF | Trachsel et al.,2019 | Serious | Not serious | Not serious | Serious | Not assessable | Low |
| Aerobic exercise + resistance training | Aerobic exercise + resistance training | LVEF | Nowak et al.,2020 | Serious | Not serious | Not serious | Serious | Not assessable | Low |
| Walking training | Usual care | LVEF | Cai et al.,2021 | Serious | Not serious | Not serious | Serious | Not assessable | Low |
| Aerobic exercise | Usual care | LVEF | Elshazly et al.,2022 | Serious | Not serious | Not serious | Serious | Not assessable | Low |
| HIIT | MICT | LVEF | Eser et al.,2022 | Serious | Not serious | Serious | Serious | Not assessable | Very low |
| Vibration training | Aerobic exercise + resistance training | LVEF | Nowak-Lis et al.,2022 | Serious | Not serious | Serious | Not serious | Not assessable | Low |
| HIIT | Aerobic exercise | LVEF | Aispuru-Lanche et al.,2023 | Serious | Not serious | Not serious | Not serious | Not assessable | Low |
| Aerobic exercise | Usual care | LVEF | Zhao et al.,2025 | Serious | Not serious | Not serious | Not serious | Not assessable | Moderate |
| Aerobic exercise + resistance training + flexibility training + passive joint movements + inspiratory muscle training + rehabilitation exercises+ balance training | Usual care | LVEF | Zhao et al.,2025 | Serious | Not serious | Not serious | Serious | Not assessable | Low |
| Cycling training | Aerobic exercise +resistance training | LVEF | Gloc et al.,2021 | Serious | Not serious | Not serious | Serious | Not assessable | Low |
| Aerobic exercise+ resistance training | Usual care | LVEF | Lima et al.,2023 | Serious | Not serious | Serious | Serious | Not assessable | Very low |
| Aerobic exercise | Aerobic exercise | LVEF | Nowak-Li et al.,2025 | serious | Not serious | Serious | Serious | Not assessable | Very low |
| Aerobic exercise | Usual care | LVEF | Izeli et al., 2016 | Very serious | Not serious | Not serious | Serious | Not assessable | Very low |
| Aerobic exercise + resistance training | Usual care | LVEF | McGregor et al., 2018 | Very serious | Not serious | Serious | Serious | Not assessable | Very low |
| Walking training | Usual care | LVEF | Ma et al., 2020 | Very serious | Not serious | Not serious | Serious | Not assessable | Very low |
| Aerobic exercise | Usual care | LVEF | Lee et al., 2021 | Very serious | Not serious | Not serious | Serious | Not assessable | Very low |
| Aerobic exercise + resistance training | Usual care | LVEF | Ma et al., 2021 | Very serious | Not serious | Not serious | Not serious | Not assessable | Very low |
| Aerobic exercise | Usual care | LVEF | Choi et al., 2022 | Serious | Not serious | Not serious | Serious | Not assessable | Very low |
| Aerobic exercise + resistance trainin | Usual care | LVEF | Hiruma et al., 2024 | Very serious | Not serious | Not serious | Very serious | Not assessable | Very low |
| Inspiratory muscle training | Usual care | LVEF | Liang et al., 2024 | very serious | Not serious | Not serious | Serious | Not assessable | Very low |
| Aerobic exercise + resistance training + flexibility Training + balance training | Usual care | LVEF | Yoon et al., 2024 | Very serious | Not serious | Not serious | Serious | Not assessable | Very low |
| Aerobic exercise | Usual care | LVEF | Hou et al., 2025 | Serious | Not serious | Not serious | Not serious | Not assessable | Very low |

**Table S3. Effects of exercise interventions on exercise performance in MI patients (n=39)**

| **Author/Year** | **Type** | **VO₂ related** | **MET** | **AT** | **HR** | **BP** | **EEC** | **Gait test** |
| --- | --- | --- | --- | --- | --- | --- | --- | --- |
| Izeli et al., 2016 (21) | AE | VO₂ max↑ | MET↑ |  | HR peak↔ | SBP peak↔ | Speed↑ |  |
| Lee et al., 2017 (23) | AE | VO₂ max↑ | MET↑ |  | HR max↔ |  | ET max↑ |  |
| Santi et al., 2018 (24) | AE | VO₂ max↑ |  |  | HR peak↔ | SBP↔ |  |  |
| Kunjan et al., 2018 (25) | AE |  |  |  |  |  | MVPA↑ | 6MWT↑ |
| Zhang et al., 2018 (27) | AE |  |  |  |  |  | ET max↑ | 6MWT↑ |
| Ma et al., 2020 (31) | AE |  |  |  |  |  |  | 6MWT↑ |
| Cai et al., 2021 (34) | AE | VO₂ max↑ | MET↑ | AT↑ |  |  | ET max↑ |  |
| Dehghani et al., 2021 (35) | AE |  |  |  | HR max↑ | SBP rest↓ |  |  |
| Gloc et al., 2021 (36) | AE | VO₂ max↑ | MET↑ |  | HR rest↔ | SBP rest↔ | ET max↑ |  |
| Bolatbekov et al., 2022 (40) | AE | VO₂ max↑ | MET↑ |  |  |  | ET max↑ | 6MWT↑ |
| Choi et al., 2022 (41) | AE | VO₂ max↑ | MET↑ |  | HR max↔ | SBP max↔ | ET max↑ |  |
| Elshazly et al., 2022 (42) | AE |  | MET↑ |  | HR rest↑ | DBP rest↑ |  |  |
| Eser et al., 2022 (43) | AE | VO₂ max↑ |  |  | HR↔ | DBP↑ | ET max↔ |  |
| Hou et al., 2024 (51) | AE | VO₂ max↑ |  |  |  |  |  |  |
| Liang et al., 2024 (54) | AE | VO₂ max↑ | MET↑ |  | HR peak↔ |  |  | 6MWT↑ |
| Aispuru-Lanche et al., 2024 (49) | AE |  |  |  | HR rest ↑ | SBP rest↓ |  |  |
| Hou et al., 2025(57) | AE | VO₂ max↑ |  |  | HR↑ | SBP↔ |  |  |
| Nowak-Li et al., 2025 (58) | AE |  | MET↔ |  | HR peak↑ | SBP peak↑ | ET max↑ |  |
| Zhao et al., 2025 (59) | AE | VO₂ max↑ | MET↑ | AT↑ |  |  |  |  |
| Kambic et al., 2022 (44) | AE |  |  |  |  |  | Arm curl test↑ | 6MWT↑ |
| Fontes-Carvalho et al., 2015 (19) | CE | VO₂ max↑ |  |  |  |  | ET max↑ |  |
| Omiya et al., 2015 (20) | CE | VO₂ max↑ |  |  | HR peak↑ |  |  |  |
| McGregor et al., 2018 (26) | CE | VO₂ max↑ |  | AT↑ | HR rest↔ | SBP↔ | Wmax↑ |  |
| Nowak et al., 2020 (32) | CE | VO₂ max↑ | MET↑ |  | HR rest↓ | SBP rest↓ | Exercise distance↑ |  |
| Gloc et al., 2021 (36) | CE | VO₂ max↑ | MET↑ |  | HR rest↔ | SBP rest↔ | ET max↑ |  |
| Ma et al., 2021 (38) | CE |  |  |  |  |  |  | 6MWT↑ |
| Wang et al., 2021 (39) | CE |  |  |  |  |  |  | 6MWT↑ |
| Kambic et al., 2022 (44) | CE |  |  |  |  |  | Arm curl test↑ | 6MWT↑ |
| Nowak-Lis et al., 2022 (45) | CE | VO₂ max↑ | MET↑ |  | HR rest↑ | SBP rest↔ | ET max↑ |  |
| Lima et al., 2023 (48) | CE | VO₂ max↑ |  | AT↔ | Basal HR↓ | SBP peak↔ | ET max↑ |  |
| Hiruma et al., 2024 (50) | CE | VO₂ max↑ |  | AT↑ | HR rest↑ | SBP rest↑ |  |  |
| Jo et al., 2024 (52) | CE | VO₂ max↑ | MET↑ |  | HR rest↔ | SBP rest↔ |  |  |
| Ksela et al., 2024 (53) | CE | VO₂ max↔ |  |  |  |  |  |  |
| Mitropouloset al., 2024 (55) | CE | VO₂ max↑ |  |  |  |  | ET max↑ |  |
| Zhao et al., 2025 (60) | CE | VO₂ max↑ | MET↑ | AT↑ |  |  |  |  |
| Trachsel et al., 2019 (29) | RE | VO₂ max↑ |  |  | HR peak↔ | SBP peak↔ | Peak Workload↑ |  |
| Nowak-Lis et al., 2022 (45) | RE | VO₂ max↑ | MET↑ |  | HR rest↓ | SBP rest↔ | ET max↑ |  |
| Liang et al., 2024 (54) | RE | VO₂ max↑ | MET↑ |  | HR peak↔ |  |  | 6MWT↑ |

**AE:** Aerobic exercise; **CE:** Compound exercise; **RE:** Resistance exercise; **VO₂ max:** Maximal oxygen uptake (mL/kg/min); **MET:** Metabolic equivalent of task; **AT:** Anaerobic threshold; **HR peak:** Peak heart Rate; **HR max:** Maximum heart rate; **HR:** Heart rate; **Basal HR:** Basal heart rate; **BP:** Blood pressure; **Peak SBP:** Peak systolic blood pressure; **SBP:** Systolic blood pressure; **SBP rest:** Resting systolic blood pressure; **DBP rest:** Resting diastolic blood pressure;**DBP:** Diastolic blood pressure; **ET max:** Maximal exercise time (min); **MVPA:** Moderate-to-vigorous-intensity physical activity (min); **W max:** Maximum workload; **6MWT:** 6-Minute walk test (m).
